# Publisher Correction: Humans rationally balance detailed and temporally abstract world models

**DOI:** 10.1038/s44271-025-00194-w

**Published:** 2025-02-05

**Authors:** Ari E. Kahn, Nathaniel D. Daw

**Affiliations:** 1 Princeton Neuroscience Institute, Princeton University, Princeton, NJ USA; 2 Department of Psychology, Princeton University, Princeton, NJ USA

**Keywords:** Human behaviour, Cognitive control, Learning algorithms

Correction to: *Communications Psychology* 10.1038/s44271-024-00169-3, published online 04 January 2025

In the version of this article initially published, ampersands were missing in equation (2), now reading “actionTowardsSample ~ 1 + rwd + sibNoRwd + onPolicy + rwd & sibNoRwd + rwd & onPolicy,” while the positive and negative terms (+, –) in the "Blockwise change analysis" section were formatted incorrectly as plus or minus operators. The equation and terms have now been amended in the HTML and PDF versions of the article.

