# Supplementary material for: Humans rationally balance detailed and temporally abstract world models
Source: Commun Psychol. 2025 Jan 4;3:1. doi: 10.1038/s44271-024-00169-3 (PMC11700031; doi:10.1038/s44271-024-00169-3)
Supplement: Supplementary file 2 — Supplementary Information [file 44271_2024_169_MOESM2_ESM.pdf]

Humans rationally balance detailed and temporally abstract world models

Ari E. Kahn[1] and Nathaniel D. Daw[1,2]

[1]Princeton Neuroscience Institute, Princeton University, Princeton, NJ, USA
[2]Department of Psychology, Princeton University, Princeton, NJ, USA

Supplement

Full regression estimates

MB Reward Only Model:

Name	Coef.	Std. Error	z	Pr(>|z|)
(Intercept)	0.036	0.01	3.5	0.0004
$reward_{t-1}$	0.43	0.026	16.0	4.5e-60

SR Reward Only Model:

Name	Coef.	Std. Error	z	Pr(>|z|)
(Intercept)	0.023	0.01	2.3	0.021
$reward_{t-1}$	0.4	0.025	16.0	1.2e-56

SR/MB Reward Only Model:

Name	Coef.	Std. Error	z	Pr(>|z|)
(Intercept)	0.031	0.01	3.1	0.0019
$reward_{t-1}$	0.43	0.026	17.0	1.2e-62

Participants Reward Only Model:

Name	Coef.	Std. Error	z	Pr(>|z|)
(Intercept)	0.13	0.022	6.1	1.2e-9
$reward_{t-1}$	0.5	0.052	9.7	4.6e-22

Full Model SR				
Name	Coef.	Std. Error	z	Pr(>|z|)
(Intercept)	0.28	0.033	8.5	2.1e-17
$reward_{t-1}$	0.13	0.067	2.0	0.046
lag1_neighborboat_reg	-0.64	0.039	-17.0	7.7e-62
lag2_neighborboat_reg	-0.22	0.027	-8.1	5.3e-16
lag3_neighborboat_reg	-0.089	0.027	-3.3	0.00098
lag4_neighborboat_reg	-0.048	0.027	-1.8	0.076
lag5_neighborboat_reg	-0.072	0.026	-2.7	0.0067
lag1_sameboat_reg	0.24	0.027	8.8	1.5e-18
lag2_sameboat_reg	0.09	0.027	3.3	0.00088
lag3_sameboat_reg	-0.022	0.027	-0.81	0.42
lag4_sameboat_reg	0.011	0.027	0.42	0.67
lag5_sameboat_reg	6.2e-5	0.026	0.0024	1.0
lag1_policy_reg	0.012	0.03	0.41	0.68
lag2_policy_reg	0.0038	0.031	0.12	0.9
lag3_policy_reg	-0.0015	0.031	-0.047	0.96
lag4_policy_reg	0.089	0.031	2.9	0.0038
lag5_policy_reg	-0.022	0.029	-0.75	0.45
lag1_oppislandavgboat_reg	-1.4	0.042	-34.0	2.8e-249
lag2_oppislandavgboat_reg	-0.52	0.041	-13.0	5.7e-38
lag3_oppislandavgboat_reg	-0.22	0.04	-5.5	3.1e-8
lag4_oppislandavgboat_reg	-0.2	0.039	-5.1	2.8e-7
lag5_oppislandavgboat_reg	-0.1	0.038	-2.7	0.0076
lag1_choice_autoreg	0.95	0.026	37.0	6.2e-299
lag2_choice_autoreg	0.83	0.026	31.0	1.5e-216
lag3_choice_autoreg	0.13	0.027	4.7	3.0e-6
lag4_choice_autoreg	0.34	0.027	13.0	5.1e-38
lag5_choice_autoreg	0.1	0.026	3.8	0.00013
$reward_{t-1}$ & *lag1_neighborboat_reg*	-0.088	0.055	-1.6	0.11
$reward_{t-1}$ & *lag2_neighborboat_reg*	-0.0027	0.054	-0.05	0.96
$reward_{t-1}$ & *lag3_neighborboat_reg*	-0.049	0.054	-0.91	0.36
$reward_{t-1}$ & *lag4_neighborboat_reg*	-0.11	0.054	-2.0	0.047
$reward_{t-1}$ & *lag5_neighborboat_reg*	-0.012	0.053	-0.23	0.82
$reward_{t-1}$ & *lag1_sameboat_reg*	0.19	0.054	3.5	0.00044
$reward_{t-1}$ & *lag2_sameboat_reg*	-0.0096	0.054	-0.18	0.86
$reward_{t-1}$ & *lag3_sameboat_reg*	-0.026	0.054	-0.49	0.62
$reward_{t-1}$ & *lag4_sameboat_reg*	0.079	0.053	1.5	0.14
$reward_{t-1}$ & *lag5_sameboat_reg*	-0.099	0.052	-1.9	0.059
$reward_{t-1}$ & *lag1_policy_reg*	0.7	0.08	8.7	3.1e-18
$reward_{t-1}$ & *lag2_policy_reg*	0.2	0.063	3.1	0.0018
$reward_{t-1}$ & *lag3_policy_reg*	0.14	0.062	2.2	0.025
$reward_{t-1}$ & *lag4_policy_reg*	0.16	0.062	2.6	0.011
$reward_{t-1}$ & *lag5_policy_reg*	0.15	0.058	2.5	0.013

Full Model MB				
Name	Coef.	Std. Error	z	Pr(>|z|)
(Intercept)	0.19	0.031	5.9	2.7e-9
$reward_{t-1}$	-0.43	0.056	-7.6	2.1e-14
lag1_neighborboat_reg	-0.68	0.04	-17.0	3.3e-64
lag2_neighborboat_reg	-0.22	0.027	-8.3	1.4e-16
lag3_neighborboat_reg	-0.15	0.026	-5.6	2.0e-8
lag4_neighborboat_reg	-0.027	0.026	-1.0	0.3
lag5_neighborboat_reg	-0.026	0.026	-1.0	0.31
lag1_sameboat_reg	0.38	0.026	14.0	1.1e-46
lag2_sameboat_reg	0.11	0.027	4.2	2.2e-5
lag3_sameboat_reg	0.023	0.026	0.88	0.38
lag4_sameboat_reg	0.0011	0.026	0.043	0.97
lag5_sameboat_reg	-0.0027	0.026	-0.1	0.92
lag1_policy_reg	0.0086	0.032	0.27	0.79
lag2_policy_reg	-0.0073	0.032	-0.23	0.82
lag3_policy_reg	-0.061	0.032	-1.9	0.058
lag4_policy_reg	0.03	0.031	0.96	0.34
lag5_policy_reg	0.023	0.029	0.78	0.44
lag1_oppislandavgboat_reg	-1.5	0.039	-39.0	0.0
lag2_oppislandavgboat_reg	-0.55	0.039	-14.0	5.8e-45
lag3_oppislandavgboat_reg	-0.2	0.039	-5.2	1.8e-7
lag4_oppislandavgboat_reg	-0.19	0.038	-5.0	4.7e-7
lag5_oppislandavgboat_reg	-0.031	0.037	-0.84	0.4
lag1_choice_autoreg	0.8	0.025	32.0	4.9e-228
lag2_choice_autoreg	0.44	0.025	17.0	9.0e-68
lag3_choice_autoreg	0.065	0.026	2.5	0.011
lag4_choice_autoreg	0.11	0.025	4.3	1.9e-5
lag5_choice_autoreg	0.043	0.025	1.7	0.084
$reward_{t-1}$ & *lag1_neighborboat_reg*	0.8	0.066	12.0	9.4e-34
$reward_{t-1}$ & *lag2_neighborboat_reg*	0.5	0.053	9.6	1.1e-21
$reward_{t-1}$ & *lag3_neighborboat_reg*	0.13	0.053	2.4	0.016
$reward_{t-1}$ & *lag4_neighborboat_reg*	0.011	0.052	0.21	0.83
$reward_{t-1}$ & *lag5_neighborboat_reg*	-0.0038	0.052	-0.072	0.94
$reward_{t-1}$ & *lag1_sameboat_reg*	0.43	0.052	8.3	1.1e-16
$reward_{t-1}$ & *lag2_sameboat_reg*	0.11	0.053	2.2	0.03
$reward_{t-1}$ & *lag3_sameboat_reg*	0.16	0.053	3.0	0.0024
$reward_{t-1}$ & *lag4_sameboat_reg*	0.015	0.052	0.28	0.78
$reward_{t-1}$ & *lag5_sameboat_reg*	0.0063	0.052	0.12	0.9
$reward_{t-1}$ & *lag1_policy_reg*	-0.0028	0.067	-0.042	0.97
$reward_{t-1}$ & *lag2_policy_reg*	0.02	0.064	0.32	0.75
$reward_{t-1}$ & *lag3_policy_reg*	-0.067	0.065	-1.0	0.3
$reward_{t-1}$ & *lag4_policy_reg*	0.025	0.063	0.4	0.69
$reward_{t-1}$ & *lag5_policy_reg*	0.026	0.058	0.45	0.65

SR/MB Hybrid Model				
Name	Coef.	Std. Error	z	Pr(>|z|)
(Intercept)	0.22	0.032	7.1	1.8e-12
$reward_{t-1}$	-0.18	0.064	-2.8	0.0055
lag1_neighborboat_reg	-0.72	0.037	-19.0	6.4e-82
lag2_neighborboat_reg	-0.23	0.027	-8.6	5.7e-18
lag3_neighborboat_reg	-0.14	0.027	-5.4	7.9e-8
lag4_neighborboat_reg	-0.064	0.026	-2.4	0.015
lag5_neighborboat_reg	-0.029	0.026	-1.1	0.27
lag1_sameboat_reg	0.31	0.027	12.0	1.9e-32
lag2_sameboat_reg	0.1	0.027	3.9	9.4e-5
lag3_sameboat_reg	0.0014	0.027	0.052	0.96
lag4_sameboat_reg	0.013	0.026	0.5	0.62
lag5_sameboat_reg	0.034	0.026	1.3	0.19
lag1_policy_reg	0.031	0.03	1.0	0.3
lag2_policy_reg	0.022	0.03	0.72	0.47
lag3_policy_reg	0.047	0.031	1.6	0.12
lag4_policy_reg	0.0093	0.03	0.31	0.76
lag5_policy_reg	-0.015	0.028	-0.53	0.59
lag1_oppislandavgboat_reg	-1.5	0.041	-37.0	6.4e-292
lag2_oppislandavgboat_reg	-0.52	0.04	-13.0	4.4e-40
lag3_oppislandavgboat_reg	-0.25	0.039	-6.5	9.9e-11
lag4_oppislandavgboat_reg	-0.16	0.038	-4.2	3.2e-5
lag5_oppislandavgboat_reg	-0.078	0.038	-2.0	0.04
lag1_choice_autoreg	0.92	0.025	37.0	5.3e-295
lag2_choice_autoreg	0.55	0.026	21.0	7.8e-99
lag3_choice_autoreg	0.18	0.026	6.7	2.3e-11
lag4_choice_autoreg	0.11	0.026	4.2	2.5e-5
lag5_choice_autoreg	0.11	0.026	4.4	1.2e-5
$reward_{t-1}$ & *lag1_neighborboat_reg*	0.34	0.064	5.4	7.2e-8
$reward_{t-1}$ & *lag2_neighborboat_reg*	0.12	0.053	2.2	0.027
$reward_{t-1}$ & *lag3_neighborboat_reg*	0.076	0.053	1.4	0.15
$reward_{t-1}$ & *lag4_neighborboat_reg*	0.12	0.053	2.3	0.024
$reward_{t-1}$ & *lag5_neighborboat_reg*	0.069	0.052	1.3	0.19
$reward_{t-1}$ & *lag1_sameboat_reg*	0.32	0.053	6.0	1.8e-9
$reward_{t-1}$ & *lag2_sameboat_reg*	0.075	0.053	1.4	0.16
$reward_{t-1}$ & *lag3_sameboat_reg*	-0.0044	0.053	-0.083	0.93
$reward_{t-1}$ & *lag4_sameboat_reg*	-0.0099	0.053	-0.19	0.85
$reward_{t-1}$ & *lag5_sameboat_reg*	-0.11	0.052	-2.1	0.037
$reward_{t-1}$ & *lag1_policy_reg*	0.54	0.082	6.6	4.9e-11
$reward_{t-1}$ & *lag2_policy_reg*	0.14	0.062	2.3	0.02
$reward_{t-1}$ & *lag3_policy_reg*	0.00041	0.062	0.0066	0.99
$reward_{t-1}$ & *lag4_policy_reg*	0.13	0.06	2.2	0.028
$reward_{t-1}$ & *lag5_policy_reg*	0.021	0.057	0.38	0.71

Participants				
Name	Coef.	Std. Error	z	Pr(>|z|)
(Intercept)	0.21	0.052	4.1	3.9e-5
$reward_{t-1}$	0.00019	0.093	0.002	1.0
lag1_neighborboat_reg	-0.69	0.053	-13.0	8.2e-39
lag2_neighborboat_reg	-0.14	0.041	-3.3	0.00089
lag3_neighborboat_reg	-0.12	0.04	-2.9	0.0035
lag4_neighborboat_reg	-0.034	0.04	-0.86	0.39
lag5_neighborboat_reg	-0.12	0.04	-2.9	0.0035
lag1_sameboat_reg	0.48	0.04	12.0	2.9e-32
lag2_sameboat_reg	0.17	0.04	4.2	2.3e-5
lag3_sameboat_reg	0.05	0.04	1.2	0.21
lag4_sameboat_reg	0.1	0.04	2.5	0.012
lag5_sameboat_reg	0.022	0.04	0.54	0.59
lag1_policy_reg	-0.038	0.044	-0.85	0.4
lag2_policy_reg	-0.058	0.044	-1.3	0.19
lag3_policy_reg	0.03	0.046	0.65	0.52
lag4_policy_reg	0.0094	0.046	0.2	0.84
lag5_policy_reg	0.046	0.044	1.1	0.29
lag1_oppislandavgboat_reg	-1.6	0.061	-25.0	5.9e-142
lag2_oppislandavgboat_reg	-0.32	0.06	-5.4	6.2e-8
lag3_oppislandavgboat_reg	-0.13	0.058	-2.2	0.027
lag4_oppislandavgboat_reg	-0.13	0.058	-2.2	0.027
lag5_oppislandavgboat_reg	-0.12	0.058	-2.0	0.045
lag1_choice_autoreg	1.3	0.04	32.0	1.5e-227
lag2_choice_autoreg	0.95	0.042	23.0	1.9e-116
lag3_choice_autoreg	0.06	0.043	1.4	0.16
lag4_choice_autoreg	0.49	0.042	12.0	1.0e-30
lag5_choice_autoreg	0.057	0.041	1.4	0.17
$reward_{t-1}$ & *lag1_neighborboat_reg*	0.74	0.11	6.8	1.3e-11
$reward_{t-1}$ & *lag2_neighborboat_reg*	0.13	0.081	1.6	0.11
$reward_{t-1}$ & *lag3_neighborboat_reg*	0.018	0.08	0.22	0.83
$reward_{t-1}$ & *lag4_neighborboat_reg*	-0.067	0.08	-0.85	0.4
$reward_{t-1}$ & *lag5_neighborboat_reg*	-0.053	0.079	-0.66	0.51
$reward_{t-1}$ & *lag1_sameboat_reg*	0.12	0.08	1.5	0.15
$reward_{t-1}$ & *lag2_sameboat_reg*	-0.016	0.08	-0.2	0.84
$reward_{t-1}$ & *lag3_sameboat_reg*	-0.042	0.08	-0.52	0.6
$reward_{t-1}$ & *lag4_sameboat_reg*	0.053	0.08	0.67	0.5
$reward_{t-1}$ & *lag5_sameboat_reg*	0.021	0.079	0.26	0.79
$reward_{t-1}$ & *lag1_policy_reg*	0.38	0.091	4.2	2.5e-5
$reward_{t-1}$ & *lag2_policy_reg*	0.12	0.089	1.3	0.19
$reward_{t-1}$ & *lag3_policy_reg*	0.26	0.092	2.8	0.005
$reward_{t-1}$ & *lag4_policy_reg*	-0.11	0.091	-1.2	0.22
$reward_{t-1}$ & *lag5_policy_reg*	-0.18	0.087	-2.0	0.043

Model recovery when SR behavior is absent

We wished to verify that no aspect of our experimental design biased model recovery towards estimating SR-like behavior if not present. To explore this possibility, we simulated a set of agents whose parameters were drawn from the estimates of a variation of non-blockwise model which did not model any SR behavior, but included a single β_{MB} and β_{TD}. We simulated 100 agents, each as a unique draw from the recovered mean and variance structure of the human data, and then sought to recover β_{MB}, β_{TD}, and β_{SR}. In Fig. 1, we plot the distributions of recovered group-level mean for β_{SR} ($\mu = 0.01, \sigma = 0.073$), as well as the estimate from our recovered participant data ($\mu = 0.866$).

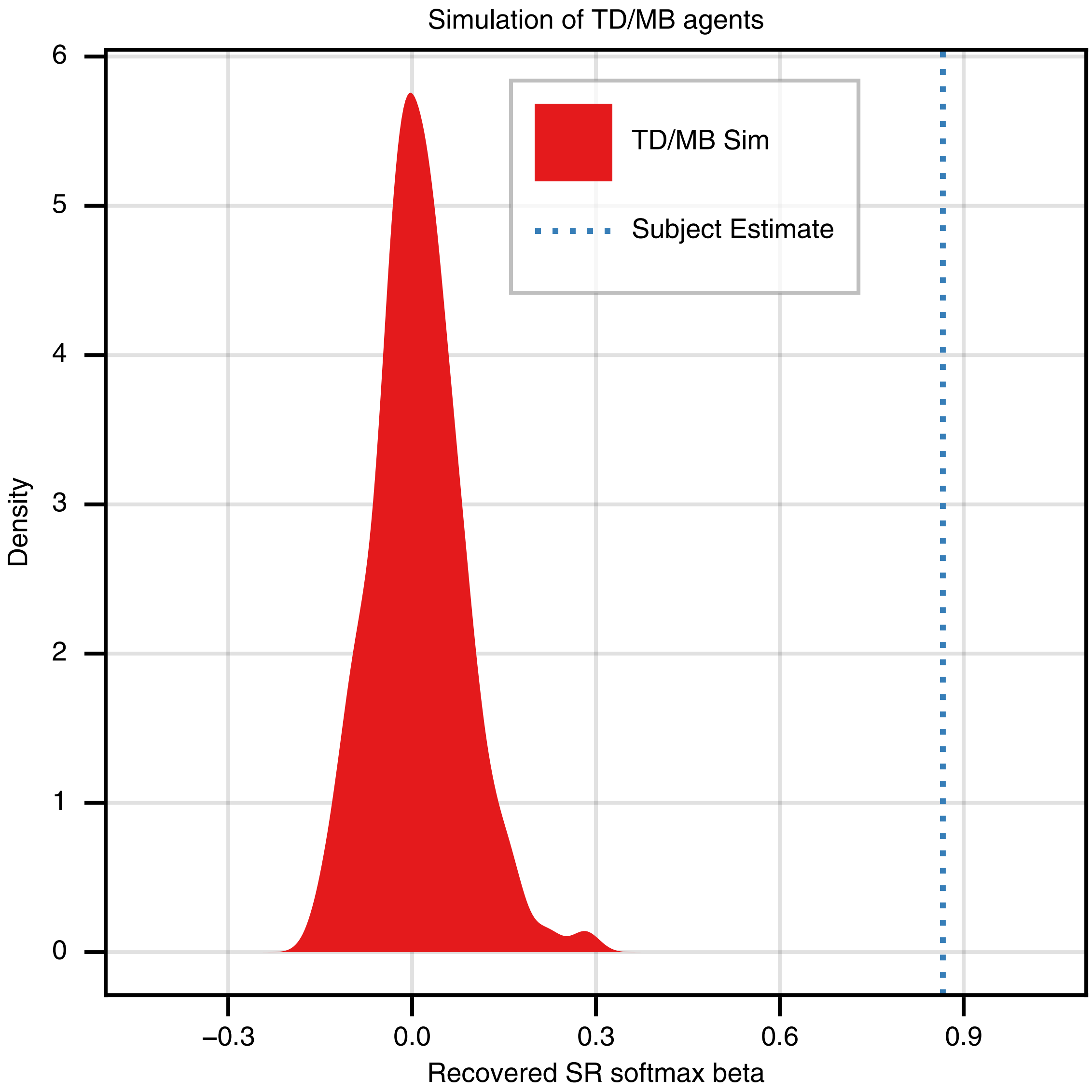

Supplementary Figure 1: **Recovery of null model simulations for SR behavior**. Distribution of estimated values of SR softmax temperature for simulated pools of TD/MB agents. The dotted line represents the value of the SR softmax temperature estimated from human participants.

Blockwise trial structure does not lead to recovery of distinct blockwise softmax parameters

A potential concern with our experimental manipulation is that the discrepancy in changed reward between our two trial types (incongruent block changes lead to a larger loss in reward when following the existing policy) may lead to a model-free shift that masquerades as a change in reliance on prior policy, and thus the MB/SR balance between blocks. To explore this possibility, we simulated a set of agents whose parameters were drawn from the estimates of our non-blockwise model: a single β_{SR}, β_{MB}, and β_{TD}. We simulated 100 agents, each as a unique draw from the recovered mean and variance structure of the human data, and then sought to recover blockwise parameters using our weighted model. We did not find a significant difference in $w_{\text{SR}}+$ and $w_{\text{SR}}-$ (Fig. 2): we found an expected difference of 0.269, 95% CI = +/- 0.57, $t_1044 = 0.92$, $p < 0.36$.

Linear RL parameter estimates for MB and SR simulations

Linear RL converges to MB behavior as $\lambda \to 0$, and to SR behavior as $\lambda \to \infty$. However, in practice, we expect to observe neither of these extremes when fitting $\log(\lambda)$ to human behavior. What values are representative of MB and SR behavior? To answer this question, we simulated agents whose parameters were drawn from the estimates of our non-blockwise-model when assuming either purely MB or purely SR behavior (that is, estimating only β_{MB} in one case and only β_{SR} in the other). We simulated 100 agents for each case, each as a unique draw from the recovered mean and variance structure of the human data, and then sought to recover a group-level estimate of $\log(\lambda)$ for each scenario. In Fig. 3, we plot the distributions of recovered group-level means for $\log(\lambda)$ (MB: $\mu = -2.347, \sigma = 0.359$, SR: $\mu = 2.742, \sigma = 0.661$, as well as the estimate from our recovered participant data ($\mu = -0.802$).

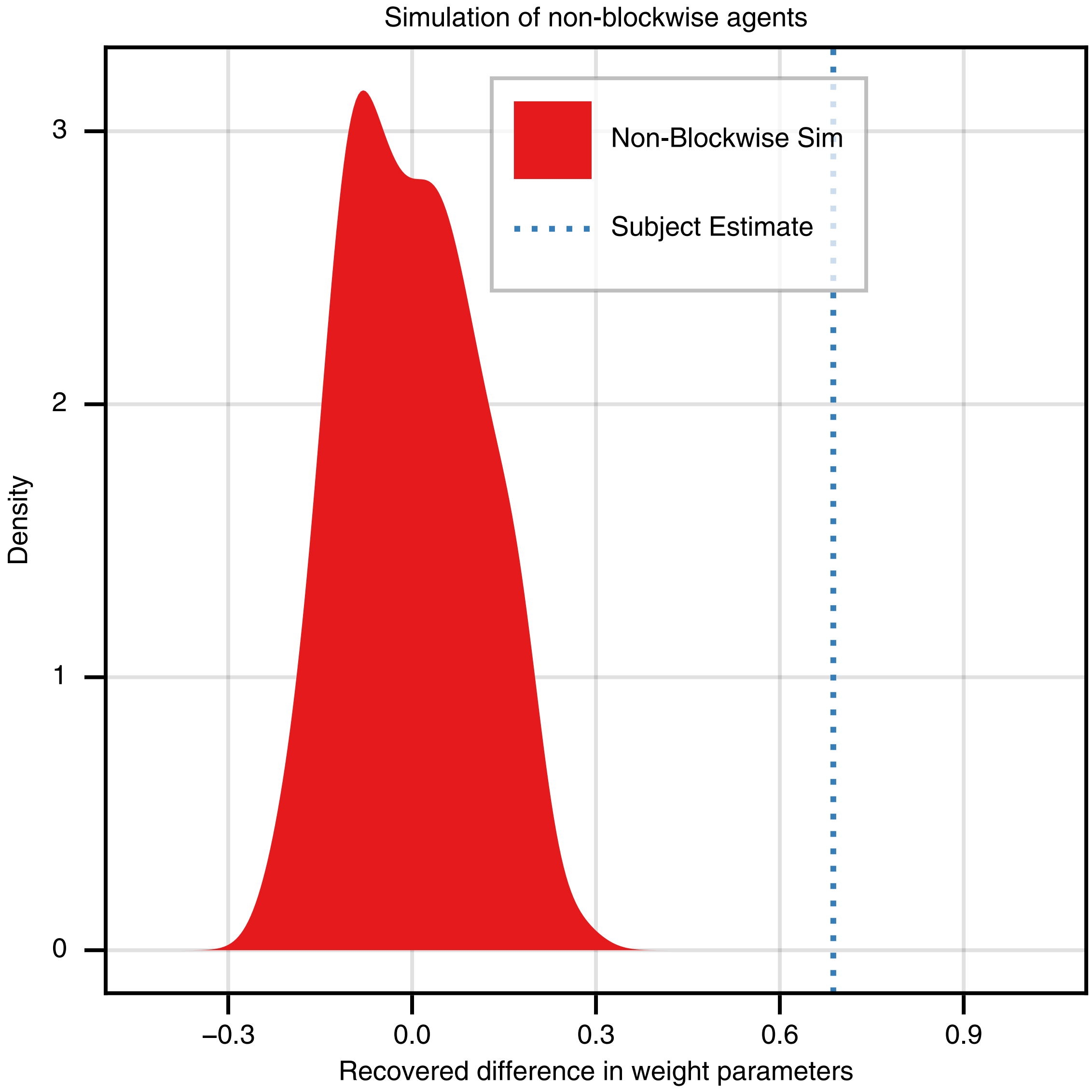

Supplementary Figure 2: **Recovery of null model simulations for blockwise behavior**. Distribution of estimated values of the differences in fit weight parameters for simulated pools of MB/SR/TD(1) agents without blockwise behavior. The dotted line represents the value of the weight difference estimated from human participants.

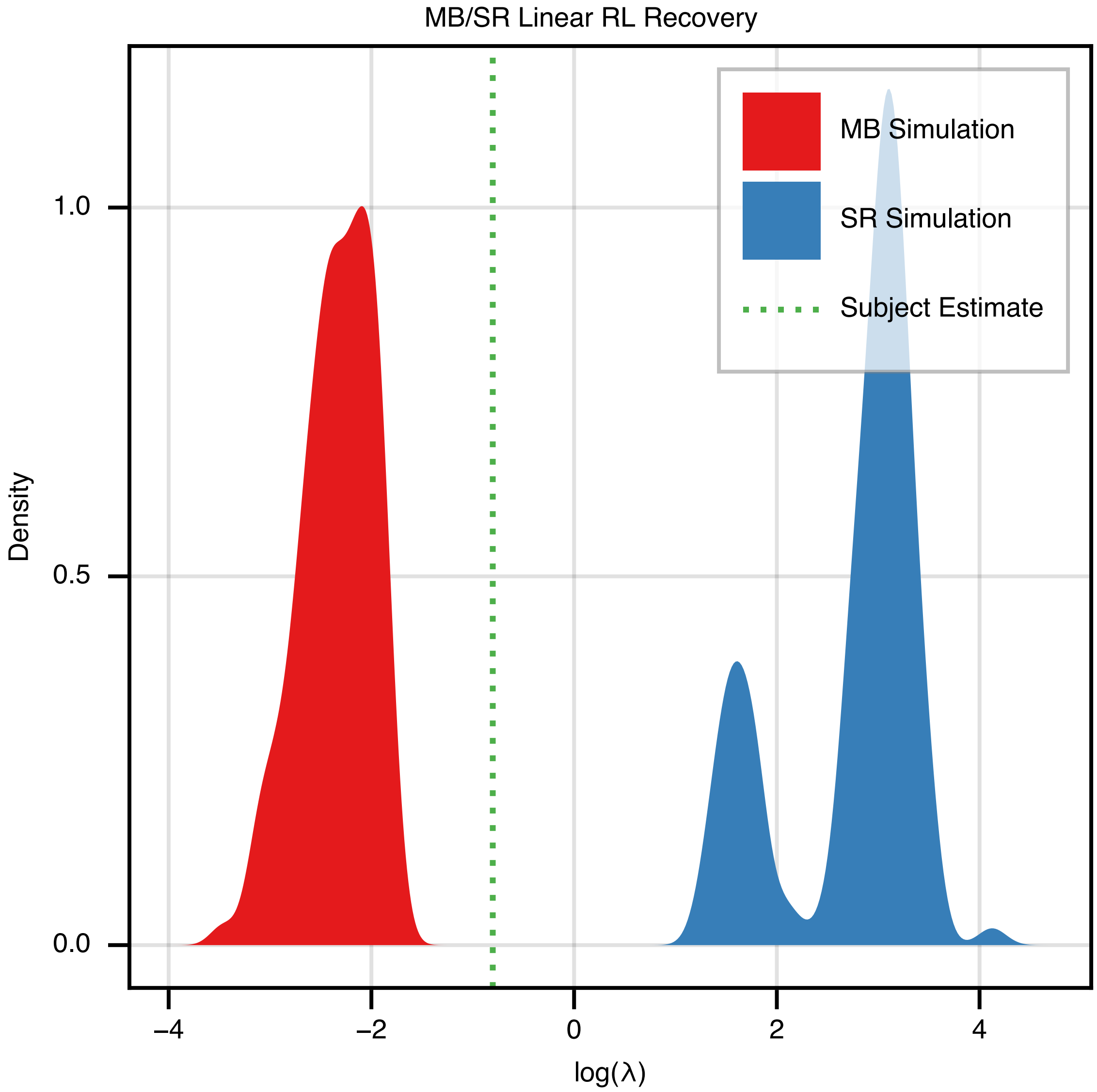

Supplementary Figure 3: **Linear RL parameter estimates**. Distribution of estimated values of log(λ) for simulated pools of either MB (red) or SR (blue) agents. The dotted line represents the value of log(λ) estimated from human participants.
