# Supplementary material for: Humans rationally balance detailed and temporally abstract world models
Source: Commun Psychol. 2025 Jan 4;3:1. doi: 10.1038/s44271-024-00169-3 (PMC11700031; doi:10.1038/s44271-024-00169-3)
Supplement: Supplementary file 3 — Reporting Summary [file 44271_2024_169_MOESM3_ESM.pdf]

nature portfolio

Corresponding author(s): Nathaniel D. Daw

Last updated by author(s): Aug 20, 2024

Reporting Summary

Nature Portfolio wishes to improve the reproducibility of the work that we publish. This form provides structure for consistency and transparency in reporting. For further information on Nature Portfolio policies, see our Editorial Policies and the Editorial Policy Checklist.

Statistics

For all statistical analyses, confirm that the following items are present in the figure legend, table legend, main text, or Methods section.

n/a	Confirmed	
☐	☒	The exact sample size (*n*) for each experimental group/condition, given as a discrete number and unit of measurement
☐	☒	A statement on whether measurements were taken from distinct samples or whether the same sample was measured repeatedly
☐	☒	The statistical test(s) used AND whether they are one- or two-sided<br>*Only common tests should be described solely by name; describe more complex techniques in the Methods section.*
☐	☒	A description of all covariates tested
☐	☒	A description of any assumptions or corrections, such as tests of normality and adjustment for multiple comparisons
☐	☒	A full description of the statistical parameters including central tendency (e.g. means) or other basic estimates (e.g. regression coefficient) AND variation (e.g. standard deviation) or associated estimates of uncertainty (e.g. confidence intervals)
☐	☒	For null hypothesis testing, the test statistic (e.g. F, t, r) with confidence intervals, effect sizes, degrees of freedom and P value noted<br>*Give P values as exact values whenever suitable.*
☐	☒	For Bayesian analysis, information on the choice of priors and Markov chain Monte Carlo settings
☐	☒	For hierarchical and complex designs, identification of the appropriate level for tests and full reporting of outcomes
☐	☒	Estimates of effect sizes (e.g. Cohen's d, Pearson's r), indicating how they were calculated

Our web collection on statistics for biologists contains articles on many of the points above.

Software and code

Policy information about availability of computer code

Data collection	All behavioral data was collected on Prolific using custom code built on top of NivTurk v1.2 and jsPysch 6.3, available at https://github.com/ariekahn/policy-sailing-task
Data analysis	Analysis was performed using Julia 1.9 and MixedModels v4.22, as well as custom expectation maximization code available at https://github.com/ndawlab/em/

For manuscripts utilizing custom algorithms or software that are central to the research but not yet described in published literature, software must be made available to editors and reviewers. We strongly encourage code deposition in a community repository (e.g. GitHub). See the Nature Portfolio guidelines for submitting code & software for further information.

Data

Policy information about availability of data

All manuscripts must include a data availability statement. This statement should provide the following information, where applicable:

- Accession codes, unique identifiers, or web links for publicly available datasets
- A description of any restrictions on data availability
- For clinical datasets or third party data, please ensure that the statement adheres to our policy

Data that support this study is available at https://osf.io/ncq34/

March 2021

Human research participants

Policy information about studies involving human research participants and Sex and Gender in Research.

Reporting on sex and gender	Participants self-reported gender as 51 male and 44 female. Two participants reported gender as "other", and three participants declined to report gender. The experiment was not designed to examine sex or gender-based differences of our main effects, nor were there any hypotheses relating to sex or gender differences.
Population characteristics	Participants self-reported age, with ages between 18 and 68 years, mean=36.19, SD=11.55
Recruitment	Participants were recruited directly through Prolific. Participants were eligible if they were fluent in English, resided in Australia, Canada, New Zealand, or the United States, and had not participated in any prior versions or pilots of the study. Subject exclusion critera were implemented through Prolific.
Ethics oversight	Institutional Review Board at Princeton University

Note that full information on the approval of the study protocol must also be provided in the manuscript.

Field-specific reporting

Please select the one below that is the best fit for your research. If you are not sure, read the appropriate sections before making your selection.

☐ Life sciences ☒ Behavioural & social sciences ☐ Ecological, evolutionary & environmental sciences

For a reference copy of the document with all sections, see nature.com/documents/nr-reporting-summary-flat.pdf

Behavioural & social sciences study design

All studies must disclose on these points even when the disclosure is negative.

Study description	Quantitative study of choice behavior employing hierarchical regression and model fitting via expectation maximization
Research sample	Online subject pool of Prolific subjects. Participants were eligible if they were fluent in English, resided in Australia, Canada, New Zealand, or the United States, and had not participated in any prior versions or pilots of the study. Subject exclusion critera were implemented through Prolific. Participants self-reported gender as 51 male and 44 female. Two participants reported gender as "other", and three participants declined to report gender. Participants self-reported age, with ages between 18 and 68 years, mean=36.19, SD=11.55.
Sampling strategy	Sample size was determined through prior online piloting
Data collection	Data were collected through online experimentation, recording reaction times and choices through keyboard input
Timing	Data were collected September 8, 2023
Data exclusions	4 participants were excluded due to outlying response times
Non-participation	18 participants returned the experiment on Prolific (no reason provided), and two dropped out due to technical reasons
Randomization	Participants were not allocated into experimental groups

Reporting for specific materials, systems and methods

We require information from authors about some types of materials, experimental systems and methods used in many studies. Here, indicate whether each material, system or method listed is relevant to your study. If you are not sure if a list item applies to your research, read the appropriate section before selecting a response.

Materials & experimental systems

n/a	Involved in the study
☒	☐ Antibodies
☒	☐ Eukaryotic cell lines
☒	☐ Palaeontology and archaeology
☒	☐ Animals and other organisms
☒	☐ Clinical data
☒	☐ Dual use research of concern

Methods

n/a	Involved in the study
☒	☐ ChIP-seq
☒	☐ Flow cytometry
☒	☐ MRI-based neuroimaging
